# The Organisms Found in the Periodontal Infections, and Their Relation to Toxaemia*Lecture, with certain additions, given at London. Manchester and Edinburgh, March and April, 1923, on behalf of the Dental Board of the United Kingdom.

**Published:** 1923-08

**Authors:** Ernest E. Glynn

**Affiliations:** M. A., M. D. Cantab, F. R. C. P. Lond. Professor of Pathology, University of Liverpool, etc.; Hon. Pathologist, Clinical Dental School, Liverpool; From the Thompson Yates Laboratory, University of Liverpool


### THE ORGANISMS FOUND IN THE PERIODONTAL INFECTIONS, AND THEIR RELATION TO THE "TOXÆMIA"*

BY ERNEST E. GLYNN, M. A., M. D. CANTAB, F. R. C. P. LOND.

Professor of Pathology, University of Liverpool, etc.; Hon. Pathologist, Clinical Dental School, Liverpool; From the Thompson Yates Laboratory, University of Liverpool

*Pathogenicity.*—The occurrence of B. fusiformis and its associated spirochætes in trench mouth, etc., has been referred to; but its pathogenicity appears to be slight, especially in the absence of spirochætes—and they seem to be absent in alveolar abscess. The bacillus is not infrequently found in metastatic abscesses in the brain and elsewhere, but usually with pyogenic cocci and other organisms. According to Hartzell (1917) there are only six cases on record of its presence in possible pure culture, but in four of these long threads grew as well as the fusiform bacilli; in one case, however, reported by Rosenow and Tunnicliff, the culture was pure. Once B. fusiformis was grown from the blood during life by Larson and Barron.

New evidence in favor of the pathogenicity of B. fusiformis has recently been brought forward by Semple, Pryce-Jones and Digby, for they found that the blood of convalescents from Vincent's angina and of certain healthy persons with fusiform bacilli in their mouths, contain "immune bodies" against the organism.

B. perfringens or B. aerogenes capsulatus, one of the gas gangrene group, is present in the normal mouth and intestine. It has been described in alveolar abscess.

B. putrificus, said to be very proteolytic but nonpathogenic, is described in normal mouths by Brailovsky-Lounkevitch and Kligler, and in putrescent pulps by Kligler. The

* Lecture, with certain additions, given at London, Manchester and Edinburgh, March and April, 1923, on behalf of the Dental Board of the United Kingdom.

Committee on the anaerobic infections of war wounds refer to the "incomplete descriptions" of this organism, and further state that war experience according to "many of the cultures of B. putrificus so-called were really mixtures" of two or three other anaerobes (M. R. C. Report 37).

Moro-Tissier Group, including B. acidophilus and B. bifidus. The first characteristic member of this group is found in the normal mouth, but also in large numbers in carious teeth. The newly-described B. acidophilus odontolyticus of McIntosh, James and Barlow appears to be closely allied to this group. Head and Roos (1919) report B. acidophilus and B. bifidus in forty-five per cent. of deep pyorrhœal pockets or periapical lesions. The incidence and pathogenicity of the various members of this group demand further investigation; in fact, all the mouth anaerobes should be restudied with the same skill and care recently devoted to those from war wounds.

### GENERAL COMPARISON BETWEEN THE BACTERIA IN (*a*) ALVEOLAR ABSCESS, GRANULOMA AND CYST, AND (*b*) GINGIVITIS AND PYORRHEA

The chief characteristics of the first group as found by workers since 1914 and also Idman may be summarized as follows:

(1) "Sterile" cultures sometimes occur, viz., in nineteen out of Hartzell and Henrici's series of chronic abscesses (Goadby, p. 62); in four out of fifty-five chronic abscesses (Gilmer and Moody), and four out of nine cysts, but in none of fifteen granulomata (Berwick). Anaerobic cultures in every case would have diminished the number of these failures—thus Idman found a streptococcus which was an obligate anaerobe in one of his eight acute abscesses. Hartzell and Henrici sometimes employed anaerobic cultures and Gilmer and Moody occasionally; Berwick did not —in his primary cultures at any rate.

It is most important to remember that in other parts of the body, bacteria may sometimes *only* grow, from the *wall* of *chronic* abscess and not from the pus.

(2) Streptococci were nearly always present and usually in pure culture, especially in the more chronic cases. They were rarely hæmolytic and then usually in acute cases; Idman found them in two out of six acute abscesses, Hartzell and Henrici on "one or two occasions" out of eight acute and many chronic abscesses and Berwick in one of his sixteen cases and that a granuloma. Gilmer and Moody "obtained many variations from hæmolytic streptococcus . . . in the acute abscess to S. viridens in the chronic."

(3) Staphylococci were the next most frequent aerobic bacteria and relatively more common in the acute abscess. They sometimes occurred in pure culture contrary to what happens in pyorrhœa. Thus Berwick found them in nine out of twenty-four cases of abscess, etc., being pure in two out of the fifteen granulomas, and Idman in three out of eight acute abscesses being pure in one, apart from an anaerobe. Hartzell and Henrici observed that staphylococci occurred in the majority of his eight acute abscesses.

Staphylococcus aureus is rare; the white staphylocci predominates, being usually according to Hartzell the nonliquefying, nonpathogenic type.

(4) Other aerobic bacteria are very rare. Gilmer and Moody describe occasional isolated colonies of M. catarrhalis, diphtheroids, etc., while Idman found a diplococcus and a diphtheroid beside one or two other bacteria.

(5) *Anaerobes.*—Though comparatively little reliable work has been done on the obligate anaerobes on account of its difficulty, yet there is no doubt that they are more frequently found in abscess than in pyorrhœa, particularly when the pus is smelling. Idman observed them in every case, finding B. perfringens and B. fusiformis among others.

THE SOURCE OF THE INFECTING BACTERIA, PARTICULARLY THE STREPTOCOCCI

This may be from:

(1) The mouth, viz., either the permanent inhabitants or temporary invaders. They may enter (*a*) directly from the surface, as probably in gingivitis or pyorrhœa; (*b*) indirectly from an infective pulp or an adjacent periodontitis, as probably in abscess, etc.

(2) Other parts of the body, viz., (*a*) the stray bacteria which occasionally enter the system and are usually got rid of, but sometimes lodge in "an area of lowered resistance"—compare the localization of tubercle bacilli in a joint after injury, or of staphylococci in a hæmatoma, causing it to suppurate (*b*) some infective focus often very distant, as a colitis, a boil, or a whitlow.

*Source in Gingivitis and Pyorrhœa.*—The evidence supporting an oral origin is very strong and may be summarized as follows: (*a*) the infected surfaces open directly into the mouth; (*b*) the remarkable similarity in the relative frequency and number of the aerobic bacteria with that found in the normal saliva, especially of the St. aureus, the lactose fermenters and the various species of hæmolytic and nonhæmolytic streptococci. The similarity in the streptococci is to a considerable extent confirmed by Hartzell and by Goadby.

It must be noted that according to Adami's "subinfection" doctrine (1899) small numbers of streptococci, or other bacteria, may sometimes pass, perhaps more or less continuously, through the damaged periodontal tissue from the mouth, and enter the blood or even distant organs. In either case they do not "necessarily multiply" (1919), but are rapidly destroyed; nevertheless toxins liberated during this destruction may produce cell degeneration followed by replacement fibrosis.

*Source in Alveolar Abscess.*—(1) The bacteria may be hæmatogenous, thus (*a*) The abscess occurs, though very rarely, around an unerupted impacted tooth (Thoma); in "noncarious apparently sound teeth" (Henrici, 1917, p. 492), or after traumatic fracture of the root of a sound tooth (Thoma); (*b*) the abscess occurs, though rarely, in vital teeth. Colyer quotes two cases described by Barker, while Hartzell reports twenty-seven cases (*Dental Review*, 1915, p. 849); some of these may be examples of the lateral alveolar abscess, etc., described by Black as due to the escape of pus from a deep pyorrhœal pocket being cut off "for any reason"; (*c*) the abscess frequently occurs in devitalized teeth; Ulrich (1915) found them in sixty-eight per cent. of over 1,000 artificially devitalized teeth—his figures are unusually high and he strongly favors hæmatogenous infection; Bumpus and Meisser (1921), of the celebrated Mayo Clinic, found that "at the apices of pulpless teeth pure cultures of a green-producing streptococcus may be isolated even when roentgenograms do not show evidence of periapical infection"; (*d*) Beretta demonstrated experimentally on dogs and guinea pigs that staphylococci, etc., when circulating in blood can localize themselves in a normal pulp chamber; they may remain there long after the blood has sterilized itself, especially in the dog, owing to the closed roots. It should be mentioned that Hartzell and Henrici (1917) "repeatedly observed that cultures from periapical tissues of healthy teeth, two or even three teeth removed from an abscess yielded streptococci." They explained this, however, by the infection spreading through the regional lymphatics.

(2) The bacteria may be *oral*. A consideration of the aerobic bacteria found in dental caries throws light upon the source of those in alveolar abscess, because it is usually consecutive to pulpitis following caries.

The pioneer work of Goadby (1893), confirmed by Kantorowicz, also by Niedergesass (1913) proved that among aerobic organisms streptococci were invariably present in the deepest layers of carious dentine; Kligler found the majority were nonhæmolytic, but Niedergesass hæmolytic.

Staphylococci were frequently present but in much smaller numbers; they were usually St. albus, very rarely St. aureus.

Now the organisms just mentioned occur in about the same relative frequency and numbers in alveolar abscesses as in caries; further, Henrici states that the streptococci he found in the former lesions were "with one or two exceptions identical with the Streptococcus salivarius of Andrewes and Horder."

It is most probable therefore that the source of the infecting aerobic bacteria in alveolar abscess, granuloma and cyst is usually *oral* and not bæmatogenous.

### THE RELATION BETWEEN THE BACTERIA FOUND IN PERIODONTAL INFECTIONS AND (*a*) THE INFLAMMATION, (*b*) THE "TOXAEMIA"

The various bacteria in periodontal inflammations have been received as regards their incidence, their relative numbers and their potential pathogenicity. It appears that the streptococci, particularly certain nonhæmolytic species and to a much lesser extent the true staphylococci, are far the most important. The evidence that these two groups, particularly the streptococci, are determining causes of alveolar abscess and granuloma is overwhelming. It remains to ascertain the part played by them in gingivitis and especially in pyorrhœa. The less important staphylococci will be taken first and their relationship to the inflammation and the "toxæmia" considered together as they are so intimately associated—the "toxæmic" effect of alveolar abscess is of course also included.

(*a*) *The Evidence of the Relationship of the Staphylococci.*—The relatively high incidence of these organisms in periodontal infections especially in acute abscesses where they are sometimes present in pure culture—apart from the possibility of anaerobes—has already been referred to. There is no need to point out that the St. albus are moderately pathogenic, for even St. epidermidis albus which liquefy gelatin very slowly, may produce stitch abscesses.

At first sight it is remarkable that St. aureus, the most pathogenic of the staphylococci and the almost invariable cause of boils, carbuncles and osteomyelitis, should be so rare in suppurative periodontitis. But this is completely explained by the comparative rarity of the organism in normal saliva.

With regard to the "toxæmia" Henrici (*Journ. Nat. Dent. Assoc.*) says "there is little data concerning the occurrence of metastatic staphylococcus infections secondary to dental lesions . . . their rarity is indicated by the paucity of the literature on the subject. Biondi's (1887) results have been confirmed by others; that is, when saliva or mixed cultures from the mouth are injected into animals, abscesses containing staphylococci sometimes develop. We have found some of our strains pathogenic, but not constantly nor even frequently." Rosenow (1921), however, isolated St. aureus from the tonsils of three patients with muscular rheumatism and produced myositis in ten rabbits by inoculating the cultures intravenously.

(*b*) The Evidence of the Relation of the Streptococci: (1) The streptococci are probably invariably present, usually in predominating numbers.

(2) Though the majority are nonhæmolytic yet S. salivarius, S. mitis and S. fæcalis are known to be pathogenic and sometimes pyogenic (see Medical Research Report, No. 79).

(3) Certain systemic lesions frequently caused by nonhæmolytic streptococci as arthritis, endocarditis, myositis and anæmia—usually secondary but sometimes pernicious[1] —are frequently associated with and may definitely follow periodontal lesions. Ulrich analysing seventy-six cases with

[1] It is quite possible that anæmia may sometimes be produced by streptococci which are nonhæmolytic in vitro. Leucocytosis, leucopenia, or lymphocytosis may occur (Goadby, Llewellyn), but I have no actual figures.

streptococci in apical "pockets" found rheumatoid conditions in 39 per cent., cardiac lesions in 36 per cent., and secondary anæmia in 33 per cent.

(4) Surgical interference with the periodontal lesion may ameliorate or even cure the systemic lesions; it may cause a temporary exacerbation from auto-inoculation (for samples, see Bumpus and Meisser, 1921); lastly it may produce fresh systemic lesions.

The enthusiasts who would ascribe all cryptic metastatic streptococcal infections to dental origin, must remember that streptococci and other organisms may enter the blood stream through lesions elsewhere, notably in the alimentary or respiratory tracts.

(5) Vaccines of streptococci from the periodontal lesion may produce local or focal reactions and may emeliorate or even cure the local or systemic lesions.

(6) The Intravenous Inoculation of Rabbits with Streptococci, specially the nonhæmolytic types from the mouth or from the periodontal lesions, often produce systemic lesions similar to those found in man, with periodontal lesions, viz., arthritis, endocarditis, myocarditis, myositis and anæmia.

The extensive observations of Hartzell and Henrici, 1914-1917, must be referred to, though they are somewhat scattered and difficult to follow. In one series, 1914-15 (see *Jour. Amer. Med. Assoc.*, 1915), they inoculated intravenously over 200 rabbits with S. viridans either from pyorrhœa or dental abscesses, the latter predominating. In a second series (see *Journ. Infect. Dis.*, 1916), they inoculated 225 rabbits with twenty-two hæmolytic strains (sixteen of S. Pyogenes and six of S. anginosus) and thirty nonhæmolytic strains, apparently viridans (seventeen S. mitis, nine S. salivarius and five S. fæcalis—Andrewes' classification). Of the nonhæmolytic strains four were from saliva, three from tonsils, nine from dental abscesses, one from pyorrhœa, the rest from fæces, endocarditis, arthritis, etc. Their conclusions may be summarized as follows:

(*a*) Mouth streptococci "whether from diseased individual or normal" are "capable of producing in rabbits lesions identical with those produced by S. viridans obtained from the lesions with rheumatism and endocarditis" (*Journ. Nat. Dent. Assoc.,* 1917, p. 494). Sometimes Aschoff-Geipel nodules, so characteristic of rheumatic myocarditis, were produced in the heart, and occasionally there was myositis of the voluntary muscle (*Journ. Infec. Dis.,* 1915).

(*b*) "Streptococci, however, from dental abscesses are more pathogenic than those from normal saliva" (*Jour. Nat. Den. Asso.,* 1917, p. 495), and "the hæmolytic streptococci were more virulent than in the nonhæmolytic but the two classes localise in the same tissues with equal frequency" (*Journ. Infec. Dis.,* 1915, p. 605).

Bumpus and Meisser (1921), examined twelve patients with pyelonephritis and cystitis and isolated "green-producing" streptococci from periapical infections only in seven, from tonsils only in three, from both in two. They injected seventy-two rabbits intravenously with the cultures, fifty-five developed focal kidney lesions and a very few myositis, arthritis or endocarditis.

Livingston (1922), when working in my laboratory, produced "very slight arthritis" in dogs by inoculating streptococci into the pulp canals of the canines .

Rosenow's observations (1915, 1919 and 1921), on "elective localisation of streptococci" must be referred to, though, perhaps, undue importance has been attached to them. Briefly he maintains that streptococci and also other organisms have a "most striking affinity or tropism for the organs or tissues from which they are isolated" (1915). In other words, streptococci from rheumatic fever are more likely to produce rheumatism when inoculated into animals than other streptococci; and streptococci from appendicitis or gastric ulcer respectively.

Rosenow's work does not necessarily weaken the evidence that the oral streptococci—so predominant in periodontal infections—can produce the various kinds of metastatic lesions just alluded to. With Ashby (1921) he inoculated many rabbits intravenously with cultures of slightly hæmolytic or green streptococci from inside the tonsils, from the pyorrhœal pus or other teeth infections of twenty-seven patients who had myositis—often complicated with neuritis or arthritis; they produced myositis in about a third of the rabbits, often complicated by neuritis or arthritis respectively, when the patient had suffered from either of these conditions.

Rosenow's work, however, has been criticised by Hartzell and others.[2] Detweiler and Robinson (1916), found that "the strains of S. viridans isolated from the mouth of normal individuals are similar to those isolated from the blood of patients suffering from chronic endocarditis, and are *equally capable* of producing heart lesions in the rabbit" (italics are mine). They also point out that contrary to Rosenow's assertions such streptococci usually had no selective or organitropic properties for certain tissues.

These observations were partly confirmed by Moody (1916). He used essentially Rosenow's technique, and inoculated 178 rabbits with S. viridans from chronic alveolar abscesses of forty-nine patients; fifteen had articular rheumatism and fifteen other forms, of "systemic disease" as gastric ulcer, anæmia, myocarditis. He concluded that the experimental lesions produced in the rabbits from the patients with or without systemic disease were "identical in character," although the streptococci from the former were rather more virulent, also that "the localisation of S. viridans can not be determined wholly by some property inherent in the organism itself."

Probably the "elective localisation of streptococci" in metastatic infections of dental origin is not so frequent as Rosenow believes.

---

[2] Rosenow inoculated his rabbits with the primary broth culture. It was therefore not necessarily pure.

(7) The similarity between the inflammatory reactions, in the periodontal lesions and those in the human systemic lesions or the animal experimental lesions.

(*a*) The histological picture of the periodontal inflammation is usually that of the subacute or chronic type. It is characterized by the presence of lymphocytes, fibroblasts and especially plasma cells, *i. e.*, a large oval cell with a round eccentric nucleus and a ''cart-wheel'' arrangement of chromatin; polymorphs are few unless there is much suppuration.

As a rule this picture is very similar to that found in the arthritis, myocarditis, etc., occurring first in man—often with periodontal lesions, second in rabbits—after the experimental inoculation of the streptococci from the healthy mouth or the periodontal lesions (Hartzell and Henrici, *Journ. Nat. Dent. Assoc.*, 1917, p. 497; Thoma, p. 74). This is strong evidence that the streptococci are pathogenic in all three.

In view of the possibility that certain spirochætes may also be pathogenic in periodontal infections, it is interesting that Stewart observed the presence of plasma cells in all five cases of cancrum oris in which he demonstrated spirochætes.

(*b*) The demonstration by the microscope of the streptococci in the living portions of the inflamed periodontal tissue is in my experience and that of many others often very difficult. They are so scanty that it may be necessary to examine many sections, or as Rosenow suggests to incubate the tissues for a few hours before fixation, in order that the cocci may multiply *in situ* (*Journ. Nat. Dent. Assoc.*, 1919, p. 988). The same difficulties, however, usually occur in demonstrating microscopically the streptococci in the animal experimental or human lesions.

This scantiness does not mean that the streptococci are not the chief, perhaps the only, bacteria producing the inflammation. It is sometimes very difficult to demonstrate tubercle bacilli in histological sections of a chronic tuberculous gland or gonococci in a case of gonorrhœal salpingitis—in which, by the way, plasma cells are very numerous.

An interesting paper by Turner and Drew (1918), must now be mentioned. They state that "portions of gum removed from chronic cases (of pyorrhœa) have invariably shown the presence of bacteria, the most frequent being diphtheroids, streptococci and staphylococci," that "sections of granulomata . . . have invariably shown the presence of organisms" and lastly that in advanced cases of pyorrhœa organisms "are often present in very large numbers" in the bone.

The give numerous photographs of the bacteria in the soft tissues; none show streptococci and one staphylococci; but many show diphtheroids which is rather remarkable. Of course it is not easy to judge from photographs, but some of them sugges that the diphtheroids are in necrotic debris or pus *on* the periodontal tissue. Others suggest that they are *in* tissues which are dead, owing to the apparent absence of an inflammatory reaction. In other words, the diphtheroids may be acting not pathogenically but as harmless saprophytes. However, the authors grew a diphtheroid, viz., a B. septus (B. coryzæ contagiosæ) from one patient with pyorrhœa, and his serum "gave a marked complement fixation to it," evidence that the bacillus was producing a pathogenic effect. The part played by diphtheroids requires further investigation; but judging by recent observations upon war wounds the great majority are either not pathogenic, or only slightly so (Adami, 1918).

### BACTERIOLOGICAL EVIDENCE THAT STREPTOCOCCI ARE NOT THE PRIMARY OR SPECIFIC CAUSE OF PYORRHOEA AND GINGIVITIS

The fact that streptococci are probably invariably present in these lesions and usually the predominating aerobic organism fulfills one of Koch's postulates. But if they were the *specific* cause it should be possible to fulfil another postulate and produce the disease experimentally in healthy animals—especially as house fed dogs are liable to pyorrhœa. No one has apparently succeeded in doing this by inoculating either streptococci or other organisms into the peridental tissues; Howe recently experimented upon guinea-pigs but without result.

On the other hand if the "tissue resistance" is lowered, gingivitis and even pyorrhœe may arise spontaneously. Thus Hartzell and Henrici (*Journ. Nat. Dent. Assoc.*, 1917), produced pyorrhœa in cats by placing wire ligatures round the necks of their teeth. They also fed cats on calomel causing mercurial stomatitis with loosening of the teeth and abscess of the jaw; streptococci were grown from the abscesses and from the cervical glands. They did not, however, examine the pus for spirochætes which have been found in the mercurial stomatitis of man by Kranz and Schlossberger (1921).

Jackson and Moody produced scurvy with gingivitis in guinea-pigs by modifying their diet; they found streptococci in the bone lesions and in the mouth as well as in the mouth of normal guinea-pigs. The Mellanbys, by feeding puppies on a diet deficient in vitamin A, produced "abnormal development of the tissues at the gingival margin" and of "the periodontal membrane." Goadby attaches considerable importance to these results, pointing out that hypertrophis gingivitis is common in children and frequently give rise to suppurative periodontitis. Alveolar abscess is easily produced. Hartzell and Henrici cut off the crowns of teeth in cats thus exposing the pulp to saliva, and produced abscesses containing streptococci in pure culture.

The demonstration of definite immunity reactions between the patient's blood and the bacteria, or spirochætes found in the periodontal lesions would throw valuable light upon their probable pathogenicity. Unfortunately for technical reasons, this is difficult or impossible, especially in the case of the spirochætes.

*Conclusion.*—In spite of the difficulties raised in the last paragraphs there is no doubt that the streptococci are the chief pathogenic aerobic organisms in periodontal infections.

It may be suggested now that the *oral* streptococci can not be so pathogenic, since all mouths contain them. The same difficulty, however, confronts us in other parts of the body. Pneumococci are frequently present in the nasopharynx, but only some develop pneumonia. Streptococci and colon bacilli are always present in the appendix, but streptococcal or colon appendicitis is not universal. It is the old problem of the seed and the soil. In gingivitis and pyorrhœa the primary change is mainly in the soil, *i. e.,* a ''lowering of tissue resistance,'' possibly sometimes an increase in the virulence of the streptococci, but probably not the advent of a *specific* streptococcus.

But once the resistance is lowered and perhaps inflammation established streptococci—particularly the nonhæmolytic oral streptococci—are the chief bacteria, which, if they do not cause the gingivitis or pyorrhœa, at least perpetuate and often aggravate it.

Streptococci are almost invariably the only organisms producing the associated ''toxæmia,'' or focal systemic infections.

We are still ignorant of all the factors lowering the resistance and especially of their relative importance; also whether the change begins in the gingival margin or the bony alveolus (see Fleischmann and Gottlieb, 1921). Heredity, increasing age, various forms of traumatism, lack of vitamins, certain spirochætes, and the endocrine glands, play or may possibly play a part. We do not really know!

The Cure, or better, the Prevention, of periodontal infections is of great national importance; it is a goal for scientific dentistry. This goal can only be reached by Research—skilled, patient, organized and co-ordinated Research. Let us try to see, not wait to see!—*The Dental Surgeon.*

